# Adipo-Myokine Modulation in Obesity: Integrative Effects of Spinach Thylakoids and Functional Training in Men with Obesity: A Randomized Controlled Clinical Trial

**DOI:** 10.3390/nu18030509

**Published:** 2026-02-02

**Authors:** Omid Razi, Asrin Shafei, Mehri Abdi, Behnam Saeidi, Parvin Farzanegi, Nastaran Zamani, Maryam N. ALNasser, Keyvan Hejazi, Abdullah Almaqhawi, Ayoub Saeidi, Rashmi Supriya, Hassane Zouhal

**Affiliations:** 1 Department of Exercise Physiology, Faculty of Physical Education and Sports Science, Razi University, Kermanshah 6714414971, Iran; omid.razi.physio@gmail.com; 2 Ergonomics Department, University of Social Welfare and Rehabilitation Science, Tehran 19857-13834, Iran; 3 Department of Physical Education and Sport Sciences, Faculty of Humanities and Social Sciences, University of Kurdistan, Sanandaj 6617715175, Iran; 4 Department of Research and Development, Razi Vaccine and Serum Research Institute, Agricultural Research, Education and Extension Organization (AREEO), Karaj P.O. Box 31975/148, Iran; 5 Department of Sport Physiology, Sari Branch, Islamic Azad University, Sari 48164-194, Iran; parvin.farzanegi@gmail.com; 6 Department of Biology, Faculty of Science, Payame Noor University, Tehran 19569, Iran; 7 Department of Biological Sciences, College of Science, King Faisal University, Al-Ahsa 31982, Saudi Arabia; malnasser@kfu.edu.sa; 8 Department of Sport Sciences, Faculty of Sport Sciences, Hakim Sabzevari University, Sabzevar 96179-76487, Iran; 9 Department of Family and Community Medicine, College of Medicine, King Faisal University, Al Ahsa 31982, Saudi Arabia; 10 Department of Sports and Health Sciences, Faculty of Arts and Social Sciences, Academy of Wellness and Human Development, Hong Kong Baptist University, Kowloon Tong 999077, Hong Kong SAR, China; 11 Centre for Health and Exercise Science Research, Hong Kong Baptist University, Kowloon Tong 999077, Hong Kong SAR, China; 12 International Institute of Sport Sciences (2I2S), 35000 Rennes, France

**Keywords:** high-intensity functional training, thylakoid, myokines, obesity

## Abstract

**Objective**: This study evaluated the effects of a 12-week High-Intensity Functional Training (HIFT) program combined with thylakoid supplementation on plasma adipo-myokine levels (Decorin, Myostatin, Follistatin, Activin A, and TGF-β1) in men with obesity. Secondary outcomes included anthropometric indices, lipid profiles, and insulin resistance markers. **Methods**: Sixty men with obesity (age: 27.6 ± 8.4 years; BMI: 32.6 ± 2.6 kg/m^2^) were randomly assigned to four groups (n = 15 per group): Placebo (PG), Supplement (SG), HIFT + placebo (TPG), and HIFT + supplement (TSG). To ensure robustness against the 27% attrition rate, statistical analyses included both per-protocol and intention-to-treat (ITT) models. HIFT was performed for 3 sessions/week (Borg scale: 15–17). **Results**: Following Bonferroni correction for multiple endpoints, repeated-measures ANOVA showed significant Time × Group interactions for most adipo-myokines and metabolic markers. Both training groups (TPG and TSG) demonstrated improvements in body composition and insulin sensitivity compared to PG (*p* < 0.05). While no significant differences were observed between TPG and TSG for systemic metabolic markers, preliminary data suggested that thylakoid supplementation might provide modest complementary modulations in specific myokines (e.g., decorin and follistatin). However, these observed trends did not reach clinical superiority over exercise alone in the broader metabolic profile. **Conclusions**: Twelve weeks of HIFT is an effective primary driver for modulating the adipo-myokine network in obese men. Although thylakoid supplementation showed potential for selective complementary effects on certain myokines, these findings are exploratory given the small sample size. The clinical significance and long-term complementary value of thylakoid-exercise interactions require further validation in larger, more diverse cohorts.

## 1. Introduction

The significant rise in obesity prevalence over recent decades has contributed to a broad spectrum of metabolic and inflammatory disorders. These conditions, by altering biochemical pathways, substantially increase the risk of developing neurodegenerative diseases [1]. This multifaceted metabolic disorder is no longer viewed merely as an energy imbalance [2] but as a primary driver of systemic morbidity and premature mortality [2,3,4,5,6]. Beyond weight gain, obesity is characterized by chronic low-grade inflammation and is fundamentally linked to the dysregulation of the Transforming Growth Factor-beta (TGF-β) signaling pathway, a key regulator of cellular growth and metabolic function. High levels of TGF-β, particularly through the SMAD pathway, promote pathological adiposity and contribute significantly to the pathogenesis of type 2 diabetes and cardiovascular complications [7,8,9].

Members of the TGF-β, including myostatin, follistatin, and activin A, play crucial roles in maintaining energy homeostasis. Myostatin acts as a negative regulator of muscle growth and promotes fat accumulation [10,11], whereas its antagonist, follistatin, enhances muscle mass and improves insulin sensitivity [12,13]. Similarly, decorin influences muscle differentiation by inhibiting myostatin and TGF-β, while also exhibiting anti-diabetic properties [14,15]. Conversely, Activin A is often elevated under inflammatory conditions, driving the proliferation of adipocyte precursors and exacerbating obesity-related metabolic dysfunction [16]. Myokines, secreted by skeletal muscle during physical activity, exert diverse endocrine effects that can counteract these disturbances [17].

High-intensity functional training (HIFT) has emerged as an effective modality to significantly modulate the myokine profile and reduce body fat through intense muscular stimulation [18]. Alongside physical activity, nutritional interventions such as thylakoid supplementation—chlorophyll-containing membranes from green leaves—have gained interest for their anti-obesity effects. Thylakoids primarily function by delaying fat digestion, which promotes the secretion of satiety hormones like GLP-1 and suppresses ghrelin [19,20]. However, the evidence regarding thylakoid efficacy remains complex and sometimes contradictory. While many clinical trials report significant weight loss and appetite suppression [21,22,23], recent studies focusing on active or well-trained populations have occasionally yielded inconsistent findings, with several investigations demonstrating no statistically significant changes in glycemic parameters or long-term lipid profiles compared to placebo groups [24,25]. These inconsistencies suggest that the metabolic benefits of thylakoids might be attenuated in individuals with higher baseline physical activity or different metabolic demands, necessitating further exploration of their effects in specific clinical contexts [26]. Given that HIFT directly impacts muscle-derived myokine secretion and thylakoids target systemic inflammation and appetite regulation, a selective complementary influence between these two interventions is highly plausible. Despite this, there is a lack of integrative research exploring their combined impact on the TGF-β family profile. Therefore, this study aimed to investigate the effects of HIFT and thylakoid supplementation on key biomarkers (decorin, follistatin, myostatin, TGF-β1, and Activin A) in sedentary men with obesity.

## 2. Materials and Methods

### 2.1. Subjects

**Figure 1:**
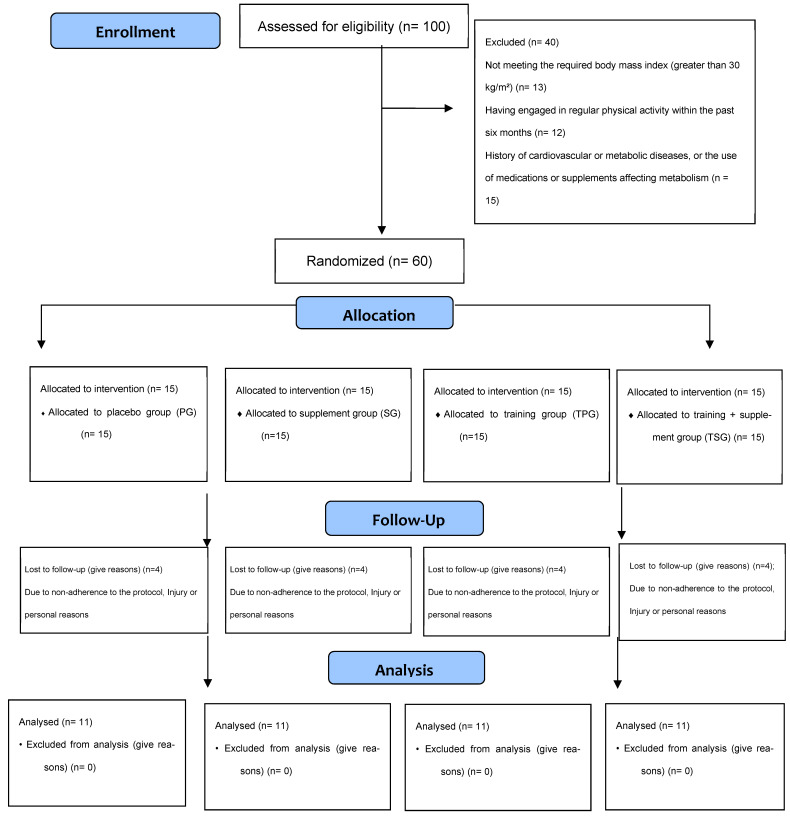
The details of the participant registration, screening, and random assignment process.

In this study, out of an initial 100 obese male volunteers, 60 were deemed eligible and participated after being screened based on inclusion and exclusion criteria. The remaining 40 volunteers were excluded from the study for primary reasons such as not meeting the required body mass index (greater than 30 kg/m^2^), having engaged in regular physical activity within the past six months, a history of cardiovascular or metabolic diseases, or the use of medications or supplements affecting metabolism. Ultimately, the eligible participants were divided into four equal groups of fifteen. After screening, 60 eligible participants were enrolled in the study. These participants were randomly assigned to one of four groups (n = 15 per group): the placebo group (PG), the supplement group (SG), the training group (TPG), and the training + supplement group (TSG). During the 12-week study period, sixteen participants from various groups withdrew from the study due to medical reasons, work-related difficulties, or a lack of interest in continuing the research. This resulted in 44 participants, on whom the final analyses were conducted (n = 11 per group) (Figure 1). Following this, 44 participants were ultimately selected (mean age: 27.6 ± 8.4 years; mean height: 168.4 ± 2.6 cm; mean body mass: 95.7 ± 3.8 kg; mean BMI: 32.6 ± 2.6 kg/m^2^).

Inclusion criteria for the study included a body mass index (BMI) greater than 30 kg/m^2^, no participation in regular physical activity within the past six months, no history of cardiovascular, metabolic, or endocrine diseases, and no alcohol consumption. Exclusion criteria for this study were defined as follows: (1) participation in regular exercise programs within the past 6 months; (2) use of medications or supplements influencing lipid or glucose metabolism; (3) smoking; and (4) habitual or excessive alcohol consumption. The latter was strictly enforced to eliminate potential confounding effects on hepatic metabolism, systemic inflammation, and adipo-myokine secretion, thereby ensuring the internal validity of the metabolic assessments. Furthermore, individuals with physical disabilities or those using prescribed medications and supplements that could interfere with muscle and adipose tissue metabolism were excluded. All participants underwent a physical examination conducted by a physician and a clinical exercise physiologist during the initial visit. Written informed consent was obtained from all participants, and they completed the physical activity readiness questionnaire (PAR-Q) [27]. Study procedures were explained to all participants at this time. The Research and Ethics Committee of the Islamic Azad University—Damghan Branch approved all study procedures (Ethics code: IR-IAU.DAMGHAN.REC.1401-034). All procedures were conducted in accordance with the latest revision of the Declaration of Helsinki [28].

### 2.2. Experimental Design

One week prior to the intervention, participants attended a familiarization session where all procedures were detailed, and baseline anthropometric and body composition assessments were performed. Participants were then randomly assigned to one of four equal groups (n = 11 per group): Placebo (PG), Spinach Thylakoid (SG), Training + Placebo (TPG), and Training + Spinach Thylakoid (TSG). To ensure balanced allocation, permuted block randomization was employed using an online generator with a block size of eight (each containing two assignments per group). To prevent selection bias, allocation was managed by an independent research assistant not involved in participant screening. The sequence was concealed in opaque, sealed envelopes, opened only after final enrollment and baseline assessments were completed.

Following baseline measurements, the TPG and TSG commenced a 12-week HIFT program (3 sessions/week), while the PG and SG maintained their habitual lifestyles. All assessments were conducted at a consistent time of day under controlled environmental conditions (20 °C; 55% humidity). Post-test measurements were performed 48 h after the final session. To minimize confounding variables, participants were instructed to maintain a standardized diet for 48 h preceding both baseline and post-intervention assessments.

### 2.3. Body Composition

Body mass and height were assessed using a calibrated scale and stadiometer (Seca, Hamburg, Germany). Body composition, including fat-free mass (FFM) and fat mass (FM), was measured using a multi-frequency bio-impedance analyzer (Medigate Company Inc., Wonju, Gangwon-do, South Korea), which has been previously validated against dual-energy X-ray absorptiometry (DXA) in obese populations. To ensure measurement validity and control for hydration status, participants were instructed to: (1) fast for at least 8 h; (2) avoid strenuous exercise for 24 h prior to testing; (3) void their bladder immediately before measurement; and (4) maintain normal hydration levels in the preceding 48 h. All measurements were conducted in the morning to minimize diurnal variations in body water distribution. These standardized conditions were strictly followed to enhance the reliability of the BIA-derived metrics.

### 2.4. Preparation of Spinach Thylakoids and Placebo

Thylakoid membranes were isolated from fresh baby spinach leaves (*Spinacia oleracea*), sourced from Tabriz, Iran, during the spring of 2020. The extraction process was performed on an experimental scale following standardized protocols previously established by Emerk et al. and others [29,30,31]. Briefly, the procedure involved homogenization of the leaves, filtration through a 20 μm polyester mesh, and pH-dependent precipitation at the isoelectric point (pH 4.7) to isolate the thylakoid fraction. After repeated washing and neutralization to pH 7.0, the collected precipitate was freeze-dried to obtain a stable, green thylakoid powder. This streamlined approach ensured the preservation of the membranes’ integrity while adhering to validated extraction methods.

The placebo group received 5 g/day of pharmaceutical-grade corn starch (obtained from Rouz Darou, Tehran, Iran), an inert and non-allergenic substance widely used in the pharmaceutical industry. To ensure the integrity of the double-blind design, the corn starch was physically modified to achieve a consistency, scent, and color nearly identical to the spinach-extracted thylakoid powder; specifically, it was colored green using edible food coloring and flavored with kiwi fruit essence. Both the thylakoid powder and the modified corn starch were then packaged in identical, opaque, and tasteless sachets. This rigorous masking procedure ensured that participants and investigators—including field researchers, outcome assessors, and the statistician—remained strictly blinded to the treatment assignments until the final data analysis was completed.

The thylakoid supplement was prepared on an experimental scale following the validated large-scale isolation method described by Emek et al. [31]. To ensure the reproducibility and potency of the intervention, the final thylakoid batch was characterized by key compositional indices. Each 5 g dose contained a standardized total chlorophyll concentration of approximately 28–32 mg/g of dry weight, determined via spectrophotometry, which serves as a primary marker for thylakoid membrane density [29,31]. The preparation maintained the biological integrity of the chloroplast membranes, a crucial factor for inhibiting pancreatic lipase/co-lipase activity and delaying fat digestion. Furthermore, based on the characterization protocols of Montelius et al. [30], the supplement was verified to be free from significant macromolecular impurities, ensuring a consistent purity profile across the 12-week trial.

Participants consumed the contents of one sachet dissolved in a glass of water 30 min before lunch. To ensure compliance, packages were coded and distributed monthly by a third party who was not involved in any other aspects of the study. A supplement consumption chart was provided to each participant as a reminder. This chart was to be returned at each visit to monitor compliance. To maximize protocol adherence, a multi-faceted monitoring strategy was employed. Participants received daily text message reminders and weekly phone calls throughout the 12-week period. Furthermore, compliance with the thylakoid supplementation was objectively verified by collecting and counting the remaining sachets during each clinic visit. In accordance with the study protocol, a participant was considered adherent and included in the final analysis only if they consumed 80% of the assigned supplements. For 12 weeks, participants consumed 5 g/day of a thylakoid-rich Thylakoid supplement or a matching placebo 30 min before lunch. The selection of a 5 g/day thylakoid dose was primarily based on previous clinical evidence demonstrating its efficacy in modulating appetite and promoting weight loss [32]. While we acknowledge that the external validity of this dosage has been predominantly established in female populations or specific clinical conditions like PCOS, the current study aims to bridge this gap by evaluating its effectiveness in men with obesity. Although gender-specific differences in body composition and hormonal profiles could potentially influence the magnitude of the response, the shared mechanisms of thylakoids in delaying fat digestion and enhancing satiety signals provide a robust rationale for this dosage in the current cohort.

### 2.5. Training Protocols

The HIFT program comprised a total of 36 sessions, each lasting up to 60 min. This study utilized the CrossFit methodology as the HIFT program. All HIFT sessions were supervised by a certified CrossFit trainer. The first two sessions served as an introduction to common movements utilized in HIFT, including exercises such as squats, deadlifts, presses, jerks, barbell, dumbbell, and medicine ball cleans, pull-ups, and kettlebell swings. No additional workouts were performed on days one and two. Starting from day three, each HIFT class followed a standardized structure:

1. Warm-up (10–15 min): Included stretching and dynamic movements to prepare the body for exercise.
2. Technique and Movement Practice (10–20 min): Focused on refining movement technique and practicing specific exercises.
3. Workout of the Day (WOD) (5–30 min): The core component of each session, which was performed at vigorous intensity relative to each participant’s individual fitness level. To ensure the standardization and control of exercise intensity across participants, a combined approach was used. Primarily, exercise intensity was monitored using the 6 to 20 borg rating of perceived exertion (RPE) scale. Participants were instructed to maintain their effort within the 15–17 range (corresponding to hard to very hard) during the main component of the WOD. Additionally, certified CrossFit trainers continuously supervised the participants’ performance and apparent effort. This was to ensure that each individual trained at their maximal safe effort, relative to their fitness level and individually scaled movements. This method allowed us to maintain a consistent relative exercise intensity at a vigorous level for all participants, despite differences in individual capabilities. Progression was achieved by increasing the number of repetitions and reducing rest intervals every four weeks, while maintaining the target intensity of 80–90% of maximum heart rate. Specific functional movements were progressed from basic to more complex variations as participants’ technical proficiency improved.

WODs incorporated a variety of exercise modalities, including aerobic (e.g., running, jumping rope), body mass (e.g., pull-ups, squats), and weightlifting (e.g., front squats, kettlebell swings). The CrossFit training template [33] was utilized to constantly vary the workout structure, employing single, couplet, or triplet modalities performed for time, repetitions, or body mass. All body mass and movements were individually prescribed and recorded for each HIFT participant [26]. Depending on the specific WOD structure, data such as time to completion, rounds and repetitions completed, body mass utilized, and any necessary modifications from the programmed workout were also recorded for each participant. Average completion times for each WOD, as well as the total average WOD time per week, were calculated for the entire HIFT group.

To ensure high reproducibility, the specific structure of the Workout of the Day (WOD) was standardized across three modalities: metabolic conditioning (M), gymnastics (G), and weightlifting (W). Representative examples of the WODs performed during the intervention are now detailed in Table 1. These included various formats such as ‘AMRAP’ and ‘For Time’ protocols. For instance, a typical triplet WOD consisted of a 400 m run (M), 15 pull-ups (G), and 10 deadlifts at 60% of 1 RM (W). All movements were scaled to the participants’ individual fitness levels while maintaining a target RPE of 15–17. Detailed records of repetitions, loads, and completion times were maintained to ensure progressive overload throughout the 12-week period.

### 2.6. Safety Protocols and Progressive Adaptation Phase

To ensure participant safety and gradual adaptation to the HIFT program, a rigorous multi-stage protocol was implemented. Prior to participation, all volunteers underwent medical screening by a physician and a clinical exercise physiologist, including the completion of the physical activity readiness questionnaire (PAR-Q) [27]. The program commenced with a two-session ‘On-Ramp’ preparatory phase focused on foundational movement techniques (e.g., squats, deadlifts, and presses) without high-intensity loads. Throughout the intervention, all sessions were supervised by a certified trainer who utilized ‘scaling’ techniques to individualize exercise intensity, load, and volume based on each participant’s daily fitness level. This standardized yet scalable approach ensured that the ‘vigorous’ intensity remained within safe physiological limits for an obese, sedentary population.

### 2.7. Nutrient Intake and Dietary Analysis

To assess dietary intake accurately and ensure no changes occurred in habitual eating patterns, participants were required to complete three-day food records (consisting of two weekdays and one weekend day) at two stages: pre-test and post-test [34]. To enhance precision, all participants received training from a nutritionist on how to correctly record food portion sizes. The raw data were analyzed using Diet Analysis Plus software, version 10 (Cengage, Boston, MA, USA), to determine total energy intake and the distribution of macronutrients, including carbohydrates, proteins, and fats. To guarantee adherence to their usual diet throughout the 12-week period, weekly telephone calls were made to the participants, and they were instructed to avoid any intentional dietary changes or the use of fat-burning supplements. Statistical analyses presented in Table 2 confirmed that nutritional intake was consistent between groups at the start of the study, and no significant changes were observed over time (*p* > 0.05). In addition to monitoring caloric intake, the precise dietary composition—including the distribution of carbohydrates, proteins, saturated fats, and daily cholesterol levels—was analyzed using Diet Analysis Plus software. To prevent any confounding bias in the results, participants were instructed to maintain their habitual consumption patterns of high-cholesterol foods throughout the study period. Weekly monitoring confirmed a high level of adherence to their usual diets. This rigorous control of dietary variables ensures that the observed changes in metabolic and biochemical parameters are strictly attributable to the training intervention and supplementation.

### 2.8. Blood Markers

All experimental procedures were conducted under standardized conditions between 8:00 and 10:00 a.m. to minimize circadian variations. To ensure a stable baseline and enhance reproducibility, fasting blood samples (after a 12 h overnight fast) were collected from the antecubital vein at the following time points: 48 h before the first training session (to establish baseline values) and 48 h after the final training session at week 12. The samples were drawn into EDTA-containing tubes, centrifuged at 3000 rpm for 10 min at 4 °C, and subsequently stored at −70 °C. Plasma concentrations of follistatin (Cat. “No.”. DFN00), myostatin (Cat. “No.”. DGDF80), and TGF-β (Cat. “No.”. DB100B) were measured using ELISA kits from R&D Systems (USA). The sensitivities for these kits were 83, 5.32, and 15.4 pg/mL, respectively, with intra-assay coefficients of variation (CVs) of 2.7%, 5.4%, and 2.9%. Additionally, plasma levels of decorin and activin A were determined using specific ELISA kits (DuoSet DY143 and DAC00B; R&D Systems, USA), with both intra-assay and inter-assay CVs maintained below 5% according to the manufacturer’s specifications.

To ensure laboratory precision, all myokine samples were analyzed in duplicate. To minimize batch effects, each participant’s samples were randomly assigned to the same plates. Alongside myokines, plasma total cholesterol (TC) and triglycerides (TG) were measured using enzymatic methods with kits from Man Company, while HDL and LDL levels were determined using Pishtaz Teb kits via an automated biochemical analyzer (made in USA). Blood glucose was also assessed enzymatically using Pars Azmoon kits (Iran) with a sensitivity of less than 2 mg/dL and an intra-assay CV of 1.82%. Finally, insulin levels were measured via ELISA using Saman Tajhiz Noor kits (Cat. “No.”. 58K2B1, Iran). The insulin resistance index was calculated using the Homeostatic Model Assessment for Insulin Resistance (HOMA-IR) equation (Equation (1)).

HOMA-IR = [Fasting glucose (mg/dL) × Fasting insulin (µU/mL)]/405(1)

### 2.9. Statistical Analysis

The sample size for this study was determined using G*Power software (version 3.1.9.7). An a priori power analysis was conducted for a repeated measures ANOVA (Time × Group) based on plasma Follistatin changes observed in previous HIFT and resistance training studies [35]. Assuming a large effect size (f = 0.40), a significance level (α) of 0.05, and a statistical power of 80%, the analysis initially indicated a minimum requirement of 14 participants per group. To account for a potential 10% attrition rate, 15 participants per group were originally recruited. However, due to the final analyzed sample of n = 44 (11 per group), a post hoc sensitivity analysis was performed. This analysis revealed that the study was sufficiently powered to detect a moderate-to-large effect size of f ≥ 0.33. All statistical procedures were conducted using SPSS software (Version 26). Statistical analysis was conducted using a repeated measures analysis of variance (ANOVA). To fully exploit the four-arm structure of the study and provide a deeper mechanistic insight, a 2 × 2 factorial framework was integrated into the model. This approach allowed for the systematic determination of the main effect of training (HIFT vs. no-HIFT), the main effect of supplementation (Thylakoid vs. Placebo), and the Time × Training × Supplementation interaction effect. In the case of a significant interaction, a Bonferroni post hoc analysis was performed to identify the specific locations of mean differences. The assumptions of normality and homogeneity of variances were confirmed prior to the ANOVA using the Shapiro–Wilk and Levene tests, respectively. Statistical analyses were conducted using a per-protocol approach, including only participants who completed the full 12-week intervention and all post-test assessments. Out of the initial 60 participants randomized, 44 completed the study (n = 11 per group). To address potential attrition bias, a sensitivity analysis was performed comparing the baseline characteristics of those who completed the study with those who withdrew; no significant differences were found. All participant flows, including reasons for withdrawal (e.g., non-compliance or personal reasons), are strictly documented in the CONSORT flow diagram to ensure consistency across the manuscript. In addition to *p*-values, we report the F-statistic with degrees of freedom (F (df_effect_, df_error_), partial eta squared (η^2^_p_) as the measure of effect size, and 95% confidence intervals (CI) for mean differences to ensure a robust and transparent presentation of the magnitude of change.

Finally, Pearson correlation coefficients were utilized to explore the relationships between changes (∆) in adipo-myokine levels and metabolic markers. To mitigate the risk of Type I error associated with multiple comparisons, a Bonferroni correction was applied, and the significance level for the correlation matrix was adjusted accordingly. Furthermore, the correlation analyses were selectively focused on primary outcomes to ensure a more robust physiological interpretation and to avoid spurious associations. All statistical procedures were performed using SPSS (Version 26), and a *p*-value of less than 0.05 was considered statistically significant.

## 3. Results

### 3.1. Factors Contributing to Attrition Across Experimental Groups 

Details regarding the enrollment process, randomized allocation, and participant flow are presented in Figure 1 (CONSORT Diagram); additionally, the specific reasons for subject attrition in each group have been documented separately in Table 3.

### 3.2. Participant Flow and Adherence

The 12-week intervention was completed by all 44 participants with an excellent adherence profile. To maximize protocol compliance, we implemented a rigorous monitoring system consisting of daily text message reminders and weekly follow-up phone calls. Supplementation adherence was objectively verified by counting returned sachets, showing a mean compliance rate of 94.2% (range: 82–100%). Similarly, the attendance rate for the HIFT sessions in the TPG and TSG averaged 91.5% (range: 85–100%). As all participants surpassed the predefined 80% adherence threshold, the final analysis was conducted on the full cohort of 44 individuals.

Training attendance was high throughout the intervention. Out of the 36 scheduled sessions (3 sessions/week × 12 weeks), participants in the TPG and TSG completed an average of 33.2 ± 1.8 sessions (range: 31–36 sessions), representing an attendance rate of 92.2%. This high level of participation ensures that the observed changes in adipo-myokine profiles are representative of a consistent exercise stimulus.

The safety of the participants during the supplementation period was ensured through rigorous monitoring protocols. All participants received necessary instructions regarding correct consumption methods and the identification of potential allergic symptoms. Weekly monitoring demonstrated that the thylakoid supplement was well-tolerated by men with obesity, and no serious adverse events occurred that necessitated discontinuation or withdrawal from the study. This high level of safety, coupled with a 94.2% adherence rate, underscores the potential of thylakoid supplementation as a safe nutritional intervention alongside HIFT.

According to Table 2, statistical analysis using ANOVA revealed no significant Time × Group interaction or main effects for total energy intake, macronutrient distribution (protein, carbohydrates, and fats), and dietary cholesterol among the four groups (*p* > 0.05). This stability in nutritional intake across the 12-week intervention effectively rules out the influence of dietary confounding factors on blood biochemical changes. Such consistency reinforces the rigor of our experimental controls, demonstrating that the observed improvements in metabolic markers and adipo-myokine profiles are directly attributable to the HIFT protocol and thylakoid supplementation rather than spontaneous alterations in dietary habits.

### 3.3. Adipo-Myokine Levels

The repeated measures ANOVA for all investigated adipo-myokines revealed significant main effects and interactions (Table 4). Specifically, for decorin, follistatin, myostatin, TGF-β1, and activin A, the main effects of time and group, as well as the Time × Group interaction, were statistically significant (all *p* < 0.001). To quantify the magnitude of these effects, Partial Eta Squared (η^2^_p_) was reported.

Statistical analyses revealed a significant interaction effect between time (training factor) and intervention groups (supplementation factor) on plasma decorin levels (F_3,56_ = 77.62, *p* < 0.001, η^2^_p_ = 0.806). Furthermore, the main effect of the group showed a significant statistical difference (F_3,56_ = 65.42, *p* < 0.001, η^2^_p_ = 0.778), indicating varying degrees of intervention efficacy across the four groups.

In the pairwise comparisons conducted with Bonferroni adjustment, the first comparison showed that the SG had a mean difference of 0.508 compared to the PG, which was not statistically significant at this level (*p* = 0.054; 95% CI: −0.006 to 1.02). Conversely, the TPG exhibited a significant mean difference of 1.63 relative to the PG (*p* < 0.001; 95% CI: 1.11 to 2.14). The most substantial result was observed in the TSG, which demonstrated a mean difference of 2.37 compared to the PG (*p* < 0.001; 95% CI: 1.86 to 2.88).

Repeated measures analysis of variance (ANOVA) revealed a statistically significant interaction effect of Time × Group on follistatin levels (F_3,56_ = 125.32, *p* < 0.001, η^2^_p_ = 0.870). Additionally, the main effect of the group demonstrated significant differences across the various protocols (F_3,56_ = 104.76, *p* < 0.001, η^2^_p_ = 0.849).

In the pairwise comparisons conducted to evaluate each intervention relative to the PG, the following results were obtained: the SG showed a mean difference of 53.94 compared to the PG, which was statistically significant (*p* = 0.001; 95% CI: 25.99 to 81.89). The TPG recorded a significant mean difference of 99.99 relative to the PG (*p* < 0.001; 95% CI: 72.04 to 127.94). Finally, the TSG exhibited the largest mean difference of 174.60 compared to the PG (*p* < 0.001; 95% CI: 146.65 to 202.55).

Data analysis revealed a statistically significant interaction effect between the measurement stages and the groups for the myostatin variable (F_3,56_ = 79.25, *p* < 0.001, η^2^_p_ = 0.809). Furthermore, the between-group comparison indicated a significant difference in the overall mean of this variable among the research groups (F_3,56_ = 62.67, *p* < 0.001, η^2^_p_ = 0.771).

The results of the Bonferroni post hoc test for the myostatin variable indicate that the mean scores in the PG differ significantly from all experimental groups (*p* < 0.001). Accordingly, the largest mean difference was observed between the PG and the TSG (Mean Difference = −1.859; 95% CI: −2.23 to −1.48). This was followed by the TPG (Mean Difference = −1.11; 95% CI: −1.48 to −0.73) and the SG (Mean Difference = −0.87; 95% CI: −1.24 to −0.49), respectively.

The results indicated a statistically significant interaction effect between the measurement stages and the research groups for the TGF-β1 variable (F_3,56_ = 52.29, *p* < 0.001, η^2^_p_ = 0.737). Additionally, the results regarding the between-group main effect revealed a statistically significant difference in the overall mean of this variable among the groups (F_3,56_ = 68.89, *p* < 0.001, η^2^_p_ = 0.787).

Based on the results of the Bonferroni post hoc test, the mean scores of the PG differed significantly from all intervention groups at the *p* < 0.001 level. In comparison with the SG, the PG showed a mean difference of −11.64 (95% CI: −15.37 to −7.90), which was statistically significant. For the TPG, a significant mean difference of −13.56 was recorded (95% CI: −17.29 to −9.82, *p* < 0.001). Finally, the comparison with the TSG revealed a mean difference of −19.06 (95% CI: −22.79 to −15.32), indicating a significant difference between these two groups.

A statistically significant interaction effect between time and groups was observed for the Activin A variable (F_3,56_ = 26.42, *p* < 0.001, η^2^_p_ = 0.586). Furthermore, the results for the between-group main effect indicated a statistically significant difference in the overall mean of this variable among the four study groups (F_3,56_ = 20.25, *p* < 0.001, η^2^_p_ = 0.520).

To further clarify these differences, the findings from the pairwise comparisons revealed that the mean scores of the PG differed significantly from all intervention groups as follows: the comparison between the PG and the SG showed a mean difference of −23.36, which was statistically significant (*p* = 0.001; 95% CI: −36.25 to −10.46). In the comparison with the TPG, the mean difference was estimated at −23.08, which was also statistically significant (*p* = 0.001; 95% CI: −35.97 to −10.19). Finally, the comparison with the TSG revealed the most substantial mean difference of −35.98, which was significant at the *p* < 0.001 level (95% CI: −48.88 to −23.09).

### 3.4. Anthropometric and Body Composition

Analysis of body composition variables (Table 5) showed that the HIFT protocol was the primary driver of change.

A statistically significant interaction effect between time and groups was observed for the body mass variable (F_3,56_ = 14.93, *p* < 0.001, η^2^_p_ = 0.444), BMI variable (F_3,56_ = 20.51, *p* < 0.001, η^2^_p_ = 0.524), FFM variable (F_3,56_ = 19.39, *p* < 0.001, η^2^_p_ = 0.510), and Fat percent variable (F_3,56_ = 21.78, *p* < 0.001, η^2^_p_ = 0.539) respectively. Furthermore, the results for the between-group main effect indicated a statistically significant difference in the overall mean of these variables among the four study groups.

To further analyze these differences, the findings from pairwise comparisons revealed the following results for body mass regarding the scores of the PG in contrast to the intervention groups:

For body mass the comparison between the PG and the SG showed statistically significant difference in mean scores (Mean Difference = −1.73; *p* = 0.013; 95% CI: −3.21 to −0.257). In the comparison with the TPG, a significant mean difference of −2.95 was recorded (*p* = 0.001; 95% CI: −4.43 to −1.47). Finally, the comparison with the TSG revealed a mean difference of −3.24, which was statistically significant at the *p* = 0.001 level (95% CI: −4.72 to −1.76).

The comparison between the PG and the SG showed no statistically significant difference in mean scores for BMI (Mean Difference = 0.682; *p* = 0.247; 95% CI: −1.57 to 0.21). In the comparison with the TPG, a non-significant mean difference of −0.443 was recorded (*p* = 1.00; 95% CI: −1.33 to 0.45). Finally, the comparison with the TSG revealed a mean difference of −1.11, which was statistically significant at the *p* = 0.007 level (95% CI: −2.00 to −0.21).

Based on the results of the Bonferroni post hoc test for FFM, the mean scores of the PG differed significantly from all intervention groups at the *p* < 0.001 level. In comparison with the SG, the PG showed a mean difference of 1.13 (95% CI: 0.154 to 2.121), which was statistically significant. For the TPG, a significant mean difference of 1.04 was recorded (95% CI: 0.062 to 2.02, *p* < 0.001). Finally, the comparison with the TSG revealed a mean difference of 1.68 (95% CI: 0.69 to 2.66), indicating a significant difference between these two groups.

Based on the results of the Bonferroni post hoc test for Fat percent, the mean scores of the PG differed significantly from all intervention groups at the *p* < 0.001 level. In comparison with the SG, the PG showed a mean difference of −1.37 (95% CI: −2.11 to −0.629), which was statistically significant. For the TPG, a significant mean difference of −1.83 was recorded (95% CI: −2.58 to −1.09, *p* < 0.001). Finally, the comparison with the TSG revealed a mean difference of −1.58 (95% CI: −2.33 to −0.84), indicating a significant difference between these two groups.

### 3.5. Lipid Profile and Insulin Resistance

The results indicated a statistically significant interaction effect between time and groups for the HDL variable (F_3,56_ = 70.79, *p* < 0.001, η^2^_p_ = 0.791). Furthermore, the between-group main effect revealed a statistically significant difference in the overall mean of this index among the four study groups (F_3,56_ = 15.09, *p* < 0.001, η^2^_p_ = 0.447) (Table 6).

To further analyze these differences, the findings from pairwise comparisons revealed the following results regarding the scores of the PG in contrast to the intervention groups:

The comparison between the PG and the SG showed no statistically significant difference in mean scores (Mean Difference = 0.42; *p* = 1.000; 95% CI: −1.04 to 1.88). In the comparison with the TPG, a significant mean difference of 2.69 was recorded (*p* = 0.001; 95% CI: −1.23 to 4.16). Finally, the comparison with the TSG revealed a mean difference of 2.79, which was statistically significant at the *p* = 0.001 level (95% CI: 1.32 to 4.26).

Statistical analysis of the LDL data revealed a significant interaction effect between the time factor and the research groups (F_3,56_ = 162.93, *p* < 0.001, η^2^_p_ = 0.897). In the same context, the evaluation of the between-group main effect confirmed a significant overall difference among the group means (F_3,56_ = 13.23, *p* = 0.001, η^2^_p_ = 0.415) (Table 6).

To further clarify these findings, simple effect analysis and pairwise comparisons revealed that the mean difference between the PG and the SG was not statistically significant (Mean Difference = −1.63, *p* = 1.000; 95% CI: −5.03 to 1.75). However, the comparison between the PG and the TPG indicated a significant reduction in the levels of this variable (Mean Difference = −6.14, *p* = 0.005; 95% CI: −9.54 to −2.75). Finally, the most substantial statistical difference was observed in the contrast between the PG and the TSG, which showed the largest mean difference compared to the control condition (Mean Difference = −6.30, *p* = 0.001; 95% CI: −9.69 to −2.90).

Statistical analysis of the TC data revealed a significant interaction effect between the time factor and the research groups (F_3,56_ = 609.43, *p* < 0.001, η^2^_p_ = 0.97). Furthermore, the evaluation of the between-group main effect indicated a significant overall difference in the mean of this index among the study groups (F_3,56_ = 21.40, *p* < 0.001, η^2^_p_ = 0.53) (Table 6).

To further clarify these differences, the results of the pairwise comparisons revealed that the mean scores of the PG differed significantly from all intervention groups. Specifically, a mean difference of −1.99 was observed in comparison with the SG, which was not statistically significant at the *p* = 1.000 level (95% CI: −6.31 to 2.33). Furthermore, the mean difference between the PG and the TPG was reported as −9.13, which was also statistically significant (*p* = 0.001; 95% CI: −13.45 to −4.80). Finally, the most substantial statistical difference was observed in the comparison with the TSG, with a value of −10.45 at the *p* < 0.001 level (95% CI: −14.77 to −6.13).

Statistical analysis of the TG data revealed a significant interaction effect between the time factor and the research groups (F_3,56_ = 205.45, *p* < 0.001, η^2^_p_ = 0.917). Additionally, the results for the between-group main effect confirmed a statistically significant difference in the overall mean of this variable among the groups (F_3,56_ = 42.30, *p* < 0.001, η^2^_p_ = 0.694) (Table 6).

To further clarify these findings, simple effect analysis and pairwise comparisons revealed varying results for the PG compared to the intervention groups. The mean difference between the PG and the SG (−1.48) was not statistically significant (*p* = 1.000; 95% CI: −6.28 to 3.31). However, the comparison between the PG and the TPG indicated a significant difference (Mean Difference = −11.47, *p* = 0.001; 95% CI: −16.27 to −6.67). Finally, the most substantial statistical difference was observed in the contrast between the PG and the TSG, which showed the highest mean difference, confirming the superiority of this protocol in altering triglyceride levels (Mean Difference = −14.56, *p* < 0.001; 95% CI: −19.36 to −9.77).

The results of the repeated measures analysis of variance (ANOVA) indicated a statistically significant interaction effect between time and the research groups for the FBS variable (F_3,56_ = 24.51, *p* < 0.001, η^2^_p_ = 0.568). This finding demonstrates significant differences in the trend of changes in this index across the various groups. Additionally, the results for the between-group main effect revealed a statistically significant difference in the overall mean of this variable among the four study groups (F_3,56_ = 6.89, *p* = 0.001, η^2^_p_ = 0.270).

Statistical analysis of the insulin data revealed a statistically significant interaction effect between the time factor and the research groups (F_3,56_ = 91.07, *p* < 0.001, η^2^_p_ = 0.830), indicating distinct changes in this index resulting from the different intervention protocols. Furthermore, the results for the between-group main effect confirmed a statistically significant difference in the overall mean of this variable among the groups (F_3,56_ = 83.30, *p* < 0.001, η^2^_p_ = 0.817).

To further clarify these differences, simple effect analysis and pairwise comparisons between the PG and the intervention groups revealed that the mean scores for this index differed significantly from the SG, with a mean difference of −0.764 at the *p* < 0.001 level (95% CI: −1.08 to −0.443). Additionally, the mean difference between the PG and the TPG was reported as −1.49, which was also found to be statistically significant at the *p* < 0.001 level (95% CI: −1.81 to −1.16). Finally, the most substantial statistical difference was observed in the comparison with the TSG, yielding a value of −1.65. This confirms the definitive superiority of this intervention protocol in altering insulin levels compared to the control condition at the *p* < 0.001 level (95% CI: −1.97 to −1.33).

Statistical analysis of the HOMA-IR data revealed a significant interaction effect between the time factor and the research groups (F_3,56_ = 56.87, *p* < 0.001, η^2^_p_ = 0.753), indicating distinct changes in this index resulting from the different intervention protocols. Furthermore, the results for the between-group main effect indicated a statistically significant difference in the overall mean of this variable among the four study groups (F_3,56_ = 19.77, *p* < 0.001, η^2^_p_ = 0.514) (Table 6).

To further clarify these differences, simple effect analysis and pairwise comparisons between the PC and the intervention groups revealed that the mean scores for this index did not differ significantly from the SG, with a mean difference of −0.247 at the *p* = 0.069 level (95% CI: −0.506 to 0.012). However, the mean difference between the PG and the TPG was reported as −0.601, which was found to be statistically significant at the *p* < 0.001 level (95% CI: −0.860 to −0.343). Finally, the most substantial statistical difference was observed in the comparison with the TSG, yielding a value of −0.615. This confirms the definitive superiority of this intervention protocol in improving the insulin resistance index compared to the control condition at the *p* < 0.001 level (95% CI: −0.872 to −0.356).

### 3.6. Total Correlations Between Different Variables

To investigate the potential relationships between variables, Pearson correlation analyses were performed, the results of which are presented as exploratory findings in Table 7. Pearson correlation analysis following the 12-week intervention revealed significant association patterns between plasma adipo-myokines, anthropometric indices, and metabolic profiles (Table 7). According to the findings, plasma decorin levels were significantly correlated with body fat percentage (r = −0.71, *p* < 0.01) and insulin levels (r = −0.86, *p* < 0.01). Similarly, follistatin demonstrated a significant negative correlation with fat mass (r = −0.78, *p* < 0.01) and insulin resistance. In contrast, myostatin and activin A levels were positively associated with body weight, fat percentage, and lipid indices, including LDL and triglycerides (*p* < 0.01). Furthermore, a significant positive correlation was observed between TGF-beta 1 levels and both myostatin (r = 0.87, *p* < 0.01) and insulin (r = 0.86, *p* < 0.01). These exploratory results suggest a reciprocal signaling network between skeletal muscle secretions and systemic metabolic status in men with obesity, although these associations do not necessarily imply causal relationships.

### 3.7. Total Correlations Between Adipo-Myokines and Metabolic Markers

To evaluate the potential relationships between measured variables, a Pearson correlation analysis was conducted on the total study population (n = 60), the results of which are detailed in Table 7. Following the 12-week intervention, a robust signaling network was identified between plasma adipo-myokines, anthropometric indices, and metabolic profiles.

According to the findings, plasma decorin levels exhibited significant and strong negative correlations with body fat percentage (r = −0.751, *p* < 0.01), insulin levels (r = −0.887, *p* < 0.01), and triglycerides (r = −0.838, *p* < 0.01). Similarly, follistatin demonstrated a potent inverse association with insulin resistance (HOMA-IR: r = −0.838, *p* < 0.01) and total cholesterol (r = −0.850, *p* < 0.01). In contrast, myostatin and activin A were positively associated with body weight (r = 0.757 and r = 0.599, respectively; *p* < 0.01) and LDL-C (r = 0.749 and r = 0.636; *p* < 0.01). Furthermore, a significant positive correlation was observed between TGF-β1 and both myostatin (r = 0.907, *p* < 0.01) and body fat percentage (r = 0.807, *p* < 0.01).

### 3.8. Exploratory Analysis of the HIFT Group (PG)

In the PG, exploratory correlation analysis revealed significant association patterns among anthropometric and metabolic markers. Body mass and fat mass were positively associated with BMI (r = 0.73 and r = 0.68, respectively; *p* < 0.05). A robust positive correlation was observed between glucose levels and HOMA-IR (r = 0.90, *p* < 0.001). Conversely, Fat-Free Mass (FFM) showed an inverse relationship with insulin (r = −0.67, *p* = 0.022). Furthermore, significant negative correlations were identified between LDL levels and both Activin A (r = −0.67, *p* = 0.023) and myostatin (r = −0.86, *p* = 0.001).

### 3.9. Inter-Relationships Among Myokines

The exploratory analysis revealed powerful interactions within the adipo-myokine profile. A very high positive correlation was noted between the two anabolic myokines, follistatin and decorin (r = 0.914, *p* < 0.01). Conversely, follistatin showed a strong inverse relationship with its antagonist, myostatin (r = −0.912, *p* < 0.01). Additionally, decorin levels were negatively linked to TGF-β1 (r = −0.845, *p* < 0.01), while myostatin and activin A demonstrated a significant positive inter-relationship (r = 0.857, *p* < 0.01).

### 3.10. Exploratory Analysis of the Thylakoid Group (SG)

Exploratory analysis within the SG highlighted several significant associations, primarily centered around glucose metabolism and adipo-myokines. A strong positive correlation was noted between glucose and HOMA-IR (r = 0.91, *p* < 0.001). Decorin levels exhibited significant positive associations with glucose (r = 0.84, *p* = 0.001), HOMA-IR (r = 0.70, *p* = 0.016), myostatin (r = 0.65, *p* = 0.029), and Activin A (r = 0.66, *p* = 0.026). Additionally, Activin A was positively linked to glucose (r = 0.71, *p* = 0.014), while myostatin correlated positively with insulin levels (r = 0.72, *p* = 0.011).

### 3.11. Metabolic and Anthropometric Associations

The correlation matrix highlighted that insulin levels were positively and significantly linked to all pro-inflammatory/catabolic markers, particularly myostatin (r = 0.914, *p* < 0.01) and TGF-β1 (r = 0.865, *p* < 0.01). Fat-free mass (FFM) showed favorable associations, correlating positively with decorin (r = 0.646) and negatively with insulin (r = −0.717). Moreover, HDL-C was positively associated with the decorin-follistatin axis (r = 0.750 and r = 0.724), emphasizing the link between muscle-derived secretions and improved lipid profiles.

## 4. Discussion

The findings of the present study suggest that 12 weeks of High-Intensity Functional Training (HIFT) may play a role in modulating the metabolic status of men with obesity by inducing alterations in the adipo-myokine profile. The observed increase in plasma decorin levels following this protocol is consistent with the hypothesis that intense muscular contractions serve as a potential stimulus for the secretion of this glycoprotein [36]. From a mechanistic perspective, it has been proposed that decorin might contribute to improving insulin sensitivity and modulating fasting glucose levels [14]—a trend that appears to be reflected in the reduction in the HOMA-IR index observed in our exercise groups. Furthermore, our data identified a potential inverse association between decorin and myostatin following HIFT. The concurrent reduction in myostatin levels within the training groups aligns with prior research suggesting decorin’s ability to interfere with myostatin signaling, possibly through the upregulation of the Mighty gene [37].

Consistent with the biological perspectives proposed by Guo et al. [38] muscle-derived myostatin—rather than its adipose-derived counterpart—is thought to function as a significant regulator of energy metabolism. Consequently, the increase in fat-free mass observed in our results may represent more than a structural adaptation; it potentially serves as a metabolic link that counteracts obesity-related complications by facilitating glucose homeostasis [38]. However, it is important to note that without tissue-level analysis, these systemic changes should be interpreted as indirect indicators of skeletal muscle adaptation.

Our results further indicate that the reduction in TGF-β1 levels following HIFT is congruent with evidence highlighting the involvement of the TGF-β1/Smad3 pathway in adipose tissue expansion [9]. Decorin may function as a biological antagonist to TGF-β1, potentially mitigating some of the constraints placed on fatty acid oxidation in the obese state [39]. This interaction could be a factor in the improved oxidative capacity observed, as the suppression of Smad3 signaling has been linked to the upregulation of metabolic genes such as PGC-1α [40]. Additionally, the modulation of the follistatin-myostatin axis emerged as a relevant outcome of the HIFT protocol. Elevated follistatin levels may support muscle protein synthesis pathways while providing a defense against insulin resistance by neutralizing myostatin and activin A [9,41]. The decline in activin A likely reflects a broader reduction in systemic inflammation rather than a specific muscular response alone [42].

Regarding thylakoid supplementation, our data reveal selective layers of interaction. While supplementation appeared to independently influence certain myokines like decorin and follistatin, the physiological dominance of the HIFT protocol in markers such as lipid profiles and body composition suggests that exercise remained the primary metabolic driver in this intervention. The lack of statistically significant differences between the TPG and TSG across most parameters suggests that thylakoid supplementation may provide modest, complementary anti-inflammatory benefits rather than a transformative synergistic effect [19,26].

Finally, the correlation analyses provide preliminary insights into a physiological network where certain myokines are associated with metabolic health. However, given the small sample size and the exploratory nature of these analyses, these associations should be viewed as a basis for hypothesis generation rather than definitive causal pathways. These patterns likely reflect complex biological interactions that require further investigation through more rigorous mechanistic studies and larger cohorts [43]. While the observed correlation patterns between decorin, follistatin, and fat-free mass are promising, these results should be considered as hypotheses for future research and must not be interpreted as definitive causal relationships, given the limited sample size and the exploratory nature of the analyses.

The findings related to correlation analyses and network interpretations in the present study provide valuable insights into the interrelationships between adipo-myokines and anthropometric indices. However, these results should be interpreted with caution as exploratory findings. Given that a large number of correlation tests were performed across various subgroups, there is a potential risk of Type I error inflation due to multiple testing. Therefore, rather than implying definitive causal relationships, these correlations are intended to serve as a foundation for hypothesis generation in future studies with larger sample sizes. These network patterns may reflect complex biological interactions that necessitate more rigorous mechanistic investigations.

### Limitations

Despite its valuable findings, the present study is subject to several limitations that warrant careful consideration. First, regarding the study population and statistical power, the relatively small sample size (n = 11 per group) and the specific focus on obese men may limit the generalizability of the findings to women or other populations, and could potentially contribute to an inflation of the reported effect sizes. While our a priori power analysis was based on a large effect size (f = 0.40), the final sample size (n = 44) after a 27% attrition rate may limit the statistical power to detect more subtle changes in certain secondary biomarkers. Although a post hoc sensitivity analysis confirmed that the study remained sufficiently powered to detect moderate-to-large effects, future research with larger cohorts is required to explore these associations with higher precision. Furthermore, while we employed both Per-Protocol and Intention-to-Treat (ITT) analyses to account for the attrition, the loss of participants may still introduce a degree of selection bias. Second, concerning methodological and dietary controls, dietary assessment relied on self-reported records which, despite close monitoring, may be prone to recall bias. While a 48 h dietary standardization was implemented prior to blood sampling, this relatively short window and the reliance on 3-day food records may not fully account for long-term dietary patterns or inter-individual variability. Additionally, although RPE is a validated tool for assessing intensity in functional training, the lack of objective verification through heart rate (HR) monitoring or blood lactate levels is a methodological limitation. To mitigate this, certified CrossFit trainers provided constant supervision to ensure that the participants’ physical effort and movement velocity remained consistent with the prescribed ‘vigorous’ intensity range (Borg scale: 15–17). Third, regarding biochemical and mechanistic interpretations, a major limitation is the use of plasma samples instead of skeletal muscle biopsies for myokine assessment. Since some of these proteins (e.g., activin A or TGF-\beta) can also be secreted by non-muscular tissues, their plasma concentrations reflect a total systemic response and cannot be attributed exclusively to skeletal muscle metabolism. Nevertheless, plasma measurements provide valuable insights into the endocrine role of these factors in inter-organ cross-talk. Future studies should employ biopsy techniques and analyze tissue-specific mRNA expression to explore the precise origin of these changes. Furthermore, despite high-precision laboratory measurements, biological fluctuations and technical variables inherent in ELISA kits remain a consistent feature of such biomarker studies. Finally, the extensive correlation analyses were exploratory in nature. Due to the issue of multiplicity, there is a potential risk of Type I error; therefore, despite the application of rigorous Bonferroni corrections, these associations should be viewed as a basis for hypothesis generation rather than definitive causal relationships or mechanistic pathways.

## 5. Conclusions

The present study demonstrates that 12 weeks of High-Intensity Functional Training (HIFT) constitutes a decisive strategy for remodeling the adipo-myokine network and enhancing the metabolic health of men with obesity. Our key findings confirm that this training protocol facilitates significant improvements in insulin sensitivity and body composition by recalibrating the balance between anabolic myokines (decorin and follistatin) and catabolic factors (myostatin and activin A). Regarding the combined intervention, although thylakoid supplementation did not yield a statistically significant clinical advantage over exercise alone in general markers—such as fat mass and lipid profiles—it induced specific enhancements in the expression of muscular anabolic markers. Collectively, these results underscore the centrality of HIFT as the primary interventional pillar, suggesting that supplementation serves merely as an adjunctive approach to bolster specific signaling pathways within skeletal muscle. These findings highlight the clinical necessity of targeting skeletal muscle biochemical signaling in the management of obesity.

## Figures and Tables

**Table 1:** Exercise modalities and Workout of the Day (WOD) structures.

Weeks	Modality	WOD Format	Example Content
1–4	Couplet (M + G)	AMRAP 12 min	200 m Sprint + 15 Air Squats
5–8	Triplet (M + G + W)	For Time	3 Rounds: 400 m Run, 20 Push-ups, 12 Kettlebell Swings
9–12	Chipper (Multiple)	For Time	50 Wall Balls, 40 Box Jumps, 30 Burpees, 20 Power Cleans

**Table 2:** Mean (±SD) values of nutritional intake in the four study groups.

	PG		SG		TPG		TSG	
	Pre	Post	Pre	Post	Pre	Post	Pre	Post
Energy (kcal/d)	2255 ± 67	2261 ± 86	2274 ± 111	2132 ± 150	2253 ± 127	2161 ± 167	2271 ± 177	2110 ± 186
CHO (g/d)	280 ± 12.4	282 ± 19.3	276.4 ± 77.1	260 ± 67.5	283 ± 48.6	264 ± 19.2	288 ± 18.6	261 ± 19.1
Fat (g/d)	81.2 ± 10.0	80 ± 8.8	85.5 ± 11.7	74 ± 13.2	80.4 ± 14.4	72.1 ± 13.2	79.8 ± 10.87	70.2 ± 15.3
Protein (g/d)	103 ± 11.0	105 ± 13.3	100 ± 14.5	94 ± 11.6	102 ± 17.8	92 ± 12.7	103 ± 15.5	89 ± 14.5

PG: placebo group; SG: Supplement group; TPG: Training + placebo group; TSG: Training + supplement group. Indicates significant differences compared to the Pre-values (*p* < 0.05).

**Table 3:** Reasons for participant attrition across study groups.

Group	Allocated (n)	Lost to Follow-Up (n)	Specific Reasons for Attrition
PG	15	4	2: Joint disorders; 1: New medication; 1: Personal reasons
SG	15	4	2: Low training attendance; 2: Unrelated injuries
TPG	15	4	2: Started caffeine supplements; 2: Non-compliance with pills
TSG	15	4	2: Relocation; 1: Started new exercise program; 1: Personal reasons

PG: placebo group; SG: Supplement group; TPG: Training + placebo group; TSG: Training + supplement group.

**Table 4:** Mean, standard deviations and effect size of study main variables using PP and ITT Analyses.

Variables	Group	Pre-Training Mean (SD)	Post-Training Mean (SD)	*p* Values (η^2^_p_)		
				Time	G × T Interaction	Group
Decorin (ng/mL) PP	PG (n = 11)	8.1 (0.6)	8.1(0.6)	<0.001 (0.3)	**<0.001** (0.7)	<0.001 (0.7)
	SG (n = 11)	8.0 (0.5)	9.2 (0.5)			
	TPG (n = 11)	7.9 (0.4)	11.5 (0.9)			
	TSG (n = 11)	8.0 (0.6)	12.9 (1.7)			
Decorin (ng/mL) ITT	PG (n = 15)	8.1 (0.4)	8.1 (0.4)	<0.001 (0.86)	**<0.001** (0.80)	<0.001 (0.77)
	SG (n = 15)	8.0 (0.4)	9.2 (0.4)			
	TPG (n = 15)	7.9 (0.3)	11.5 (0.8)			
	TSG (n = 15)	8.0 (0.4)	12.9 (1.4)			
Follistatin (pg/mL) PP	PG (n = 11)	1310.3 (43.7)	1286.6 (33.4)	<0.001 (0.5)	**<0.001** (0.8)	<0.001 (0.8)
	SG (n = 11)	1311.4 (41.2)	1393.4 (38.2)			
	TPG (n = 11)	1312.9 (42.8)	1484 (36.5)			
	TSG (n = 11)	1321.9 (19.6)	1624.2 (73.7)			
Follistatin (pg/mL) ITT	PG (n = 15)	1310.3 (36.9)	1286.6 (28.2)	<0.001 (0.89)	**<0.001** (0.87)	<0.001 (0.84)
	SG (n = 15)	1311.4 (34.7)	1393.3 (32.28)			
	TPG (n = 15)	1312.9 (36.2)	1483.9 (30.8)			
	TSG (n = 15)	1321.9 (16.5)	1624.1 (62.28)			
Mayostatin (pg/mL) PP	PG (n = 11)	11.8 (0.8)	12.0 (0.5)	<0.001 (0.3)	**<0.001** (0.7)	<0.001 (0.7)
	SG (n = 11)	12.0 (0.7)	10.1 (0.5)			
	TPG (n = 11)	12.1 (0.6)	9.5 (0.5)			
	TSG (n = 11)	12.0 (0.9)	8.0 (0.4)			
Mayostatin (pg/mL) ITT	PG (n = 15)	11.7 (0.6)	12.0 (0.4)	<0.001 (0.88)	**<0.001** (0.80)	<0.001 (0.77)
	SG (n = 15)	11.9 (0.6)	10.1 (0.3)			
	TPG (n = 15)	12.1 (0.5)	9.4 (0.4)			
	TSG (n = 15)	12.0 (0.7)	8.0 (0.3)			
TGF-β1 (pg/mL) PP	PG (n = 11)	90.3 (5.6)	97.7 (5.9)	<0.001 (0.4)	**<0.001** (0.7)	<0.001 (0.7)
	SG (n = 11)	86.4 (6.1)	78.4 (5.6)			
	TPG (n = 11)	86.9 (5.7)	74.0 (4.9)			
	TSG (n = 11)	88.3 (7.7)	61.6 (7.9)			
TGF-β1 (pg/mL) ITT	PG (n = 15)	90.3 (4.7)	97.6 (5.0)	<0.001 (0.65)	**<0.001** (0.73)	<0.001 (0.78)
	SG (n = 15)	86.3 (5.1)	78.3 (4.7)			
	TPG (n = 15)	86.8 (4.8)	74.0 (4.2)			
	TSG (n = 15)	88.3 (6.4)	61.5 (6.6)			
Activin A (pg/mL) PP	PG (n = 11)	250.5 (17.7)	270.2 (23.2)	<0.001 (0.2)	**<0.001** (0.5)	<0.001 (0.4)
	SG (n = 11)	243.6 (22.1)	230.5 (17.2)			
	TPG (n = 11)	262. 3 (25.9)	212.3 (22)			
	TSG (n = 11)	253.4 (24.8)	195.5 (22.1)			
Activin A (pg/mL) ITT	PG (n = 15)	250.5 (14.9)	270.2 (19.5)	<0.001 (0.48)	**<0.001** (0.58)	<0.001 (0.52)
	SG (n = 15)	243.6 (18.6)	230.4 (14.5)			
	TPG (n = 15)	262. 2 (21.8)	212.3 (18.5)			
	TSG (n = 15)	253.3 (21.0)	195.4 (18.7)			

TGF-β1: Transforming Growth Factor-beta 1, η^2^p: Partial Eta Squared (Effect Size). Data are presented as mean ± SD *p* < 0.05 indicates a significant difference. Bonferroni correction was applied to all post hoc pairwise comparisons to control for Type I error across multiple outcomes. Results reflect the Per-Protocol analysis. However, a sensitivity check using an Intention-to-Treat (ITT) model confirmed the stability of the primary outcomes despite participant attrition. Adjusted *p*-values are reported following Bonferroni adjustment.

**Table 5:** Mean, standard deviation and effect size of study variables using PP and ITT Analyses.

Variables	Group	Pre-Training Mean (SD)	Post-Training Mean (SD)	*p* Values (η^2^_p_)		
				Time	G × T Interaction	Group
Body mass (Kg) PP	PG (n = 11)	94.33 (1.82)	93.55 (2.43)	<0.001 (0.63)	**<0.001** (0.44)	<0.001 (0.37)
	SG (n = 11)	93.28 (2.61)	91.13 (2.12)			
	TPG (n = 11)	92.78 (1.89)	89.19 (2.37)			
	TSG (n = 11)	94.13 (1.90)	87.25 (2.30)			
Body mass (Kg) ITT	PG (n = 15)	94.33 (1.53)	93.55 (2.06)	<0.001 (0.70)	**<0.001** (0.51)	<0.001 (0.44)
	SG (n = 15)	93.28 (2.21)	91.13 (1.79)			
	TPG (n = 15)	92.78 (1.60)	89.19 (2.00)			
	TSG (n = 15)	94.13 (1.61)	87.25 (1.94)			
BMI (Kg/m^2^) PP	PG (n = 11)	33.08 (1.34)	32.87 (1.44)	<0.001 (0.63)	**<0.001** (0.45)	0.11 (0.14)
	SG (n = 11)	32.66 (1.37)	31.93 (0.93)			
	TPG (n = 11)	33.22 (1.07)	31.85 (1.19)			
	TSG (n = 11)	33.05 (0.75)	30.68 (0.95)			
BMI (Kg/m^2^) ITT	PG (n = 15)	33.08 (1.13)	32.87 (1.22)	<0.001 (0.70)	**<0.001** (0.52)	0.11 (0.17)
	SG (n = 15)	32.66 (1.16)	31.93 (0.78)			
	TPG (n = 15)	33.22 (0.90)	31.85 (1.01)			
	TSG (n = 15)	33.05 (0.63)	30.68 (0.81)			
FFM (Kg) PP	PG (n = 11)	27.63 (1.20)	26.54 (2.25)	<0.001 (0.46)	**<0.001** (0.43)	0.01 (0.23)
	SG (n = 11)	27.09 (1.81)	29.36 (0.92)			
	TPG (n = 11)	26.72 (1.27)	29.54 (1.5)			
	TSG (n = 11)	27.18 (1.77)	30.36 (1.2)			
FFM (Kg) ITT	PG (n = 15)	27.63 (1.01)	26.54 (1.90)	<0.001 (0.53)	**<0.001** (0.51)	0.01 (0.29)
	SG (n = 15)	27.09 (1.53)	29.36 (0.78)			
	TPG (n = 15)	26.72 (1.07)	29.54 (1.27)			
	TSG (n = 15)	27.18 (1.50)	30.36 (1.01)			
Fat percent PP	PG (n = 11)	30.09 (1.51)	30.83 (2.05)	<0.001 (0.54)	**<0.001** (0.46)	<0.001 (0.42)
	SG (n = 11)	30.10 (1.59)	28.08 (0.79)			
	TPG (n = 11)	30.36 (1.50)	26.89 (0.95)			
	TSG (n = 11)	31.13 (1.35)	26.62 (1.21)			
Fat percent ITT	PG (n = 15)	30.09 (1.27)	30.83 (1.73)	<0.001 (0.61)	**<0.001** (0.53)	<0.001 (0.49)
	SG (n = 15)	30.10 (1.35)	28.08 (0.67)			
	TPG (n = 15)	30.36 (1.26)	26.89 (0.80)			
	TSG (n = 15)	31.13 (1.14)	26.62 (1.02)			

BMI: Body Mass Index, FFM: fat-free mass, η^2^p: Partial Eta Squared (Effect Size). Data are presented as mean ± SD. *p* < 0.05 indicates a significant difference. Bonferroni correction was applied to all post hoc pairwise comparisons to control for Type I error across multiple outcomes. Results reflect the Per-Protocol analysis. However, a sensitivity check using an Intention-to-Treat (ITT) model confirmed the stability of the primary outcomes despite participant attrition. Adjusted *p*-values are reported following Bonferroni adjustment.

**Table 6:** Mean, standard deviation and effect size of lipid profiles and insulin resistance index using PP and ITT Analyses.

Variables	Group	Pre-Training Mean (SD)	Post-Training Mean (SD)	*p* Values (η^2^_p_)		
				Time	G × T Interaction	Group
HDL (mg/dL) PP	PG (n = 11)	39.34 (1.22)	38.43 (1.30)	<0.001 (0.72)	**<0.001** (0.73)	<0.001 (0.37)
	SG (n = 11)	38.88 (1.23)	39.74 (3.99)			
	TPG (n = 11)	38.66 (1.67)	44.51 (1.34)			
	TSG (n = 11)	38.56 (1.41)	44.80 (2.12)			
HDL (mg/dL) ITT	PG (n = 15)	39.34 (1.03)	38.43 (1.10)	<0.001 (0.78)	**<0.001** (0.79)	<0.001 (0.44)
	SG (n = 15)	38.88 (1.04)	39.74 (3.37)			
	TPG (n = 15)	38.66 (1.41)	44.51 (1.13)			
	TSG (n = 15)	38.56 (1.19)	44.80 (1.79)			
LDL (mg/dL) PP	PG (n = 11)	125.22 (4.47)	125.02 (4.70)	<0.001 (0.91)	**<0.001** (0.86)	<0.001 (0.34)
	SG (n = 11)	125.56 5.42)	121.41 (5.24)			
	TPG (n = 11)	126.75 (4.38)	111.20 (2.92)			
	TSG (n = 11)	127.14 (3.64)	110.50 (2.52)			
LDL (mg/dL) ITT	PG (n = 15)	125.22 (3.78)	125.02 (3.97)	<0.001 (0.93)	**<0.001** (0.89)	<0.001 (0.41)
	SG (n = 15)	125.56 4.58)	121.41 (4.43)			
	TPG (n = 15)	126.75 (3.70)	111.20 (2.47)			
	TSG (n = 15)	127.14 (3.08)	110.50 (2.13)			
TC (mg/dL) PP	PG (n = 11)	226.70 (5.27)	226.81 (5.26)	<0.001 (0.97)	**<0.001** (0.96)	0.01 (0.45)
	SG (n = 11)	227.44 (5.48)	222.09 (5.19)			
	TPG (n = 11)	227.81 (5.29)	207.44 (4.95)			
	TSG (n = 11)	227.38 (5.49)	205.23 (4.77)			
TC (mg/dL) ITT	PG (n = 15)	226.70 (4.45)	226.81 (4.45)	<0.001 (0.98)	**<0.001** (0.97)	0.01 (0.53)
	SG (n = 15)	227.44 (4.63)	222.09 (4.39)			
	TPG (n = 15)	227.81 (4.47)	207.44 (4.19)			
	TSG (n = 15)	227.38 (4.64)	205.23 (4.03)			
TG (mg/dL) PP	PG (n = 11)	242.10 (4.39)	242.81 (3.85)	<0.001 (0.90)	**<0.001** (0.89)	<0.001 (0.62)
	SG (n = 11)	245.83 (5.93)	242.05 (5.40)			
	TPG (n = 11)	244.58 (7.48)	217.39 (9.88)			
	TSG (n = 11)	242.92 (5.96)	212.85 (4.64)			
TG (mg/dL) ITT	PG (n = 15)	242.10 (3.71)	242.81 (3.25)	<0.001 (0.93)	**<0.001** (0.91)	<0.001 (0.69)
	SG (n = 15)	245.83 (5.01)	242.05 (4.57)			
	TPG (n = 15)	244.58 (6.32)	217.39 (8.35)			
	TSG (n = 15)	242.92 (5.03)	212.85 (3.92)			
FBS (mg/dL) PP	PG (n = 11)	96.44 (13.10)	90.73 (6.43)	<0.001 (0.79)	**<0.001** (0.46)	<0.001 (0.21)
	SG (n = 11)	98.99 (10.71)	84.77 (4.50)			
	TPG (n = 11)	99.27 (5.72)	74.08 (5.43)			
	TSG (n = 11)	101.63 (7.13)	71.51 (7.71)			
FBS (mg/dL) ITT	PG (n = 15)	96.44 (11.07)	90.73 (5.44)	<0.001 (0.83)	**<0.001** (0.56)	<0.001 (0.27)
	SG (n = 15)	98.99 (9.05)	84.77 (3.80)			
	TPG (n = 15)	99.27 (4.84)	74.08 (4.59)			
	TSG (n = 15)	101.63 (6.03)	71.51 (6.52)			
Insulin (µU/mL)	PG (n = 11)	18.81 (0.67)	19.12 (0.57)	<0.001 (0.83)	**<0.001** (0.78)	<0.001 (0.76)
	SG (n = 11)	18.80 (0.75)	17.60 (0.51)			
	TPG (n = 11)	18.83 (0.40)	16.12 (0.43)			
	TSG (n = 11)	19.12 (0.49)	15.51 (0.55)			
Insulin (µU/mL) ITT	PG (n = 15)	18.81 (0.56)	19.12 (0.48)	<0.001 (0.87)	**<0.001** (0.83)	<0.001 (0.81)
	SG (n = 15)	18.80 (0.63)	17.60 (0.43)			
	TPG (n = 15)	18.83 (0.34)	16.12 (0.36)			
	TSG (n = 15)	19.12 (0.41)	15.51 (0.46)			
HOMA-IR PP	PG (n = 11)	4.48 (0.70)	4.27 (0.30)	<0.001 (0.86)	**<0.001** (0.69)	<0.001 (0.43)
	SG (n = 11)	4.58 (0.46)	3.68 (0.25)			
	TPG (n = 11)	4.61 (0.24)	2.94 (0.26)			
	TSG (n = 11)	4.79 (0.38)	2.73 (0.31)			
HOMA-IR ITT	PG (n = 15)	4.48 (0.59)	4.27 (0.26)	<0.001 (0.89)	**<0.001** (0.75)	<0.001 (0.51)
	SG (n = 15)	4.58 (0.39)	3.68 (0.21)			
	TPG (n = 15)	4.61 (0.20)	2.94 (0.22)			
	TSG (n = 15)	4.79 (0.32)	2.73 (0.27)			

HDL: High-density lipoprotein, LDL: Low-density lipoprotein, TC: Total cholesterol, TG: Triglyceride, FBS: fasting blood sugar. η^2^p: Partial Eta Squared (Effect Size). Data are presented as mean ± SD. *p* < 0.05 indicates a significant difference. Bonferroni correction was applied to all post hoc pairwise comparisons to control for Type I error across multiple outcomes. Results reflect the Per-Protocol analysis. However, a sensitivity check using an Intention-to-Treat (ITT) model confirmed the stability of the primary outcomes despite participant attrition. Adjusted *p*-values are reported following Bonferroni adjustment.

**Table 7:** Pearson Correlation Matrix between Anthropometric Indices, Lipid Profiles, and Adipo-Myokines after 12 Weeks of Intervention.

Variables	Weight	BMI	FFM	FAT	HDL	LDL	TC	TG	Insulin	FBS	Decorin	Activin A	Folistatin	Myostatin	TGF-β1
Weight	1														
BMI	0.719 **	1													
FFM	−0.550 **	−0.579 **	1												
FAT	0.653 **	0.628 **	−0.711 **	1											
HDL	−0.608 **	−0.436 *	0.480 **	−0.661 **	1										
LDL	0.680 **	0.525 **	−0.603 **	0.727 **	−0.705 **	1									
TC	0.665 **	0.407 *	−0.597 **	0.710 **	−0.747 **	0.825 **	1								
TG	0.660 **	0.445 **	−0.513 **	0.640 **	−0.826 **	0.802 **	0.874 **	1							
Insulin	0.739 **	0.607 **	−0.717 **	0.813 **	−0.748 **	0.832 **	0.847 **	0.820 **	1						
FBS	0.573 **	0.388 *	−0.500 **	0.590 **	−0.664 **	0.789 **	0.751 **	0.735 **	0.821 **	1					
Decorin	−0.685 **	−0.525 **	0.646 **	−0.751 **	0.750 **	−0.811 **	−0.823 **	−0.838 **	−0.887 **	−0.724 **	1				
Activin A	0.599 **	0.489 **	−0.596 **	0.671 **	−0.578 **	0.636 **	0.724 **	0.683 **	0.814 **	0.711 **	−0.701 **	1			
Folistatin	−0.729 **	−0.533 **	0.629 **	−0.728 **	0.724 **	−0.781 **	−0.850 **	−0.808 **	−0.874 **	−0.745 **	0.914 **	−0.769 **	1		
Myostatin	0.757 **	0.587 **	−0.662 **	0.778 **	−0.707 **	0.749 **	0.806 **	0.761 **	0.914 **	0.772 **	−0.817 **	0.857 **	−0.912 **	1	
TGF-β1	0.608 **	0.592 **	−0.683 **	0.807 **	−0.605 **	0.714 **	0.720 **	0.711 **	0.865 **	0.711 **	−0.845 **	0.781 **	−0.878 **	0.907 **	1

Note: Pearson correlation coefficients calculated based on both Per-Protocol and Intention-to-Treat (ITT) datasets (n = 60) to ensure the stability of the findings. Delta indicates the change from baseline to week 12. To mitigate Type I error and control for multiple comparisons, a Bonferroni correction was applied, with the statistical significance threshold set at *p* < 0.005 for the correlation matrix. All reported correlations remained consistent across both analytical models. * Correlation is significant at the 0.05 level; ** Correlation is significant at the 0.01 level. Abbreviations: BMI: Body Mass Index; FFM: Fat-Free Mass; FAT: Body Fat Percentage; HDL-C: High-Density Lipoprotein Cholesterol; LDL-C: Low-Density Lipoprotein Cholesterol; TC: Total Cholesterol; TG: Triglycerides; FBS: Fasting Blood Glucose; HOMA-IR: Homeostatic Model Assessment for Insulin Resistance; TGF-β1: Transforming Growth Factor-beta 1.

## Data Availability

The data included in this study are available upon reasonable request.
